# Hospital and Institutional News

**Published:** 1913-11-15

**Authors:** 


### HOSPITAL AND INSTITUTIONAL NEWS.

### BRENTFORD GUARDIANS' LATEST DECISION.

At last the Brentford Union Board of Guardians has determined the appointment of a successor to Dr. E. E. Norton in the position of medical superintendent of the infirmary. Sir John Rose Bradford, in a letter to the Board, states that he has examined Dr. T. B. Cook, of St. Giles' Infirmary, Cleveland Street, W., the successful candidate in response to the second advertisement, and is of opinion that he is in sound health and physically fit for service. The Local Government Board is to be notified of Dr. Cook's appointment, subject to the Board's approval. A remarkable decision just reached by the Board is the adoption of new Standing Orders, by which meetings will be held once, instead of twice a month, and this, despite the fact that during the past year or more the meetings have been longer and less orderly than ever before. A good deal has been heard, lately, of the infirmary, where, for the want of proper provision for the isolation of infectious and septic cases, there is a risk of disease being spread rather than cured. It is not surprising, however, that affairs are in this state; a Board that cannot find good government for itself is not likely to give good government to its institutions.

### THE LADY WITH THE CLEAR HEAD.

As we publish elsewhere this week a preliminary character sketch of Florence Nightingale, as Sir Edward Cook's biography portrays it, it is interesting to recall the condition to-day of the work which she left at the call of the Crimea. That work was, of course, the superintending of the institution which is now known as The Florence Nightingale Hospital for Gentlewomen. It is characteristic that Florence Nightingale's clear head, without which her heart, so unhealthily exploited by sentimental voluptuaries, would have done more harm than good, foresaw, at that date, the need for providing for this class of patient. The needs of this class appeal more to the brains than the hearts of the hospital-subscribing public, which, it is to be hoped, with Sir Edward Cook's fine achievement in portraiture (all the more discreet because of his judicious self-concealing art), will wipe out the debt of £1,000 which is crippling this hospital's resources at the present time.

### DEATH OF THE SENIOR MEDICAL INSPECTOR OF THE LOCAL GOVERNMENT BOARD.

Dr. Richard Deane Sweeting, the senior medical inspector of the Local Government Board, died last Monday. Born in the Bahamas and educated at Malvern and Oxford, an M.D. of Dublin, he was Buxton Scholar at the London Hospital, he was called to the Bar, having gained the Holt Scholarship in Constitutional Law at Gray's Inn. He had ten years' experience as a medical superintendent at the Western Fever Hospital under the Metropolitan Asylums Board. He joined the medical department of the Local Government Board in 1890, having done previous temporary work for this department during the cholera survey of five years before. He had a wide experience of public health work and an exceptional knowledge of epidemiology, while his essay on the "Sanitation of Public Institutions" won the Howard Prize given by the Statistical Society. Oxford, Dublin, and Cambridge gave him respectively the degrees of Arts and Medicine and the Diploma of Public Health, and he was an examiner for both English Universities. When the Royal Commissions on Vaccination and on Hospitals were sitting he gave evidence before each, and his death at the early age of fifty-seven will perhaps draw attention to the fact that even high abilities like these cannot possibly cope with the amount of medical inspection which ought to be undertaken by the Local Government Board. It cannot be too often repeated that there are only two medical inspectors for Poor-Law purposes under the Board for the whole of England and Wales. High qualifications from every point of view are needed for this work, but a man cannot be in two places at once, and up-to-date Poor-Law infirmary superintendents would welcome only too gladly more medical inspections to aid them in pressing reforms on recalcitrant Guardians. But for more numerous inspections more inspectors are necessary. Now that a new senior inspector has to be appointed the point again should be pressed on the Local Government Board. Mr. Burns's attention should be called to this need by the joint action of the superintendents and associations to which they belong.

### MENTAL SUPERINTENDENT ON SWEATED ATTENDANTS.

From December 1 next a new scale of salaries comes into force at Cardiff Mental Hospital. In the discussion which accompanied this decision Dr. Edwin Goodall, the medical superintendent, said that there was no doubt that the attendants and nurses had been sweated in the past. This frank admission was evidently endorsed by the hospital committee, as the following recommendations were adopted unanimously. After urging that the applications of the attendants and nurses should be dealt with before the meeting ended, Dr. Goodall recommended an increase of £1 or £2, as the case might be, and an allowance of 9s. per week for ration-money on the annual leave. The adoption of this was promptly agreed to. The new proposals will cost £330 a year and will raise the cost of maintenance per head per week by 3½d. to 12s. 10d. per patient. Meanwhile, a sub-committee has been appointed to deal with the applications of the stokers (now presented for the fifth time) for a rise from their present pay of 29s. 9d. a week, with superannuation allowance and no deductions. A comparison of stokers' pay in seventeen mental hospitals was stated to show that in five higher pay was given and in twelve lower than at Cardiff. The gardeners and farm workers are also having similar applications considered. In favour of the stokers it may perhaps be said that once a stoker, always a stoker, is the rule, exceptions being rare enough to show that this occupation *per se* has practically no scope for improvement, though the really competent stoker is perhaps as rare as, say, the really competent single-handed domestic servant.

### MEDICAL TEACHERS AND PRIVATE PRACTICE.

The conflict between the claims of remunerative private practice and unremunerative (often completely unremunerated) teaching of students is an old story in our medical schools. Teachers of clinical subjects know that if they devote themselves to teaching they will starve; so they teach just so long as will fill up the time until they get a flourishing practice. In America they are taking steps to mend this muddle-headed system. The Johns Hopkins Medical School has received a million and a half dollars for the endowment of professorships and assistantships with such salaries as shall make them independent of private practice; and, in fact, the resignation of all outside work is to be demanded as one of the conditions attaching to the appointments. Dr. W. S. Halsted, the celebrated surgeon, has already agreed to retain his Chair under these new conditions, which aim at enabling the University teachers to devote the whole of their energies to research, investigation, and teaching. It is understood that the services of professors under this new arrangement will be available for the public in certain circumstances, but gratuitously. The experiment will be watched with interest; obviously, it is only possible where, as in the United States, the immensely wealthy endow educational schemes with the greatest liberality. The name of the William H. Welsh Endowment has been given to the benefaction.

### HOSPITAL SUNDAY AND SATURDAY COMPARED.

A suggestive light on what must be called the decline of the Hospital Sunday movement is thrown by the activity of the sister organisation, namely, Hospital Saturday. At Coventry, the Coventry and Warwickshire Hospital has received from this fund £3,850, or actually £250 more than last year. The new enlargement of the hospital is increasing the cost of maintenance by some £800 a year, and the present position is satisfactory, as Mr. E. M. Iliffe, the hospital's chairman, pointed out, in that £400 is in hand towards the increased expenses. As to the future, the Rev. J. E. Thomas has pointed out the value of the criticisms, and the spirit in which they are offered, that the workers give in addition to their support, and no better combination or augury for future prosperity could be suggested. Mr. Edgar Turrell said that the hospital's authorities were looking longingly at the derelict part of the old brewery at the back of the institution, and begged the Mayor, Col. W. F. Wyley, to remember his promise to assist in obtaining part of this ground at a future date. What can be done for Hospital Saturday can be done for Hospital Sunday. Can the clergy be content to see the workshops more persuasive of the public conscience than their own churches?

### £80,000 DIVERTED FROM PHILANTHROPY.

A Mr. C. L. Forrest, who was for many years a member of the Victorian Legislative Assembly, died in August last year leaving an estate of about £100,000. He was a bachelor, and apparently his nearest relatives were a nephew and a grand-niece. He provided for these by leaving £5,000 to each of them, and then bequeathed the rest of his estate to form a trust fund, the income from which was to be paid to "such Charities, Hospitals, Philanthropical Institutions or Objects, Free Libraries or Mechanics' Institutes, Churches, or other objects of a like or similar nature in the State of Victoria as his Trustees, in their absolute discretion, should deem fit." The validity of this large bequest has been challenged at law by the nephew aforesaid on the ground of vagueness and uncertainty, and the Chief Justice of Victoria (Sir John Madden) has given judgment that the will is void from uncertainty, so that if such decision stands this munificent gift will be lost for ever to the charities of Victoria. The general opinion has hitherto been that in will cases where things are somewhat indefinite the law seeks to get behind and carry out the intention of the testator, but in this case it would seem as if the Court had gone absolutely against that principle, and its decision is enough to make the late Mr. Forrest not merely "turn in his grave," but get out of it altogether in order to give expression to the feelings of indignation that such a judgment would have aroused in him had he been alive. A deputation, consisting of nearly 100 representatives of hospitals and other charities, waited on the State Premier on September 17 and asked (*a*) that legislation might be introduced to prevent the recurrence of a case of this kind, and (*b*) to take all steps possible to save Mr. Forrest's estate for the benefit of the charities as the testator undoubtedly intended it should be. Mr. Watt, in reply, said that Parliament naturally encouraged charitable bequests and had made them free from taxation. He had already moved in the direction of the legislation asked for which should prove remedial, but would not discuss the question of making it retrospective. However, the Attorney-General has already agreed to appeal against Sir John Madden's judgment in the Forrest case.

### A NOVEL FORM OF PRIVATE HOSPITAL.

Some little time ago a brief announcement was made in our columns concerning a novel form of private hospital just being started at Duff House, Banff, formerly the property of the Duke of Fife. A most interesting and attractively produced booklet has now been prepared giving fuller details of this unique institution, which confines its attentions to the scientific investigation and treatment of diseases of metabolism. Duff House was built about one hundred and seventy years ago from designs by the elder of the famous Adam brothers: its most striking architectural feature externally is perhaps the horseshoe staircase, somewhat reminiscent of that in the Cour des Adieux of the royal château of Fontainebleau. It stands in a magnificent park, wherein amongst other amenities is a private eighteen-holes golf course. For its new purpose the house has been fitted with every requisite for treatment and diagnosis, including $x$-rays, laboratories, baths, and up-to-date equipment of every kind. The diseases for which Duff House especially caters include diabetes, gout, obesity, anæmia, Graves's disease, dyspepsia, colitis, ulceration of stomach or intestines, and habitual constipation. Mental cases, active tuberculosis, and infective diseases are not admitted. The name of Dr. E. I. Spriggs, the head physician, is sufficient guarantee of the thoroughly genuine and scientific character of the work of the institution; for this physician had already established a name for himself as an authority on metabolism and its disorders before he left London. Dr. Spriggs, it will be remembered, was assistant physician to St. George's Hospital and to the City of London Hospital for Diseases of the Chest, and physician to the Victoria Hospital for Children. Unfortunately, the very magnificence of the accommodation and excellence of treatment provided entail fees which are beyond the average man's purse. The charge is ten guineas a week, inclusive of everything except special nursing and laundry.

### COMMISSION ON SYPHILIS TO SIT IN PRIVATE.

The first meeting of the Royal Commission on Venereal Diseases was held on Friday last week. The Commissioners decided that future meetings should be held in private, but that a summary of the evidence given at the meetings should be issued to the press every week.

### NEW WAYNFLETE PROFESSOR OF PHYSIOLOGY.

Dr. Charles Scott Sherrington, M.A., M.D., F.R.S., D.Sc., has been appointed Waynflete Professor of Physiology at Oxford. The successor of Dr. Francis Gotch is a successor in the peculiar sense that he has followed the late Dr. Gotch not merely at Oxford, but at Liverpool, where he held the Holt Professorship of Physiology after Dr. Gotch had vacated it for the Chair at Oxford. A Cambridge man he went to London, where he was lecturer in his subject at St. Thomas's Hospital, and where he became also Professor of Pathology at London University. Apart from almost innumerable honorary degrees and the gold medals which the two Royal Colleges have conferred upon him, he has several varied distinctions. When the Board of Trade appointed a committee to investigate and report on Sight Tests he was a member. The Silliman Lecture in America has been delivered by him, while his work on "The Integrative Action of the Nervous System" is a contribution of recognised importance. The Waynflete Professorship, which carries with it a Fellowship at Magdalen, was founded thirty-six years ago by the University Commissioners.

### ORTHOPÆDY IN THEORY AND AT BIRMINGHAM.

Readers of an article in this issue, entitled "Modern Orthopædics and What They Mean," will, perhaps, even better appreciate what the developments in orthopædy, new to England, do mean, and the need for introducing them, when the contentions are studied in the light of the proposals emanating from the Royal Orthopædic and Spinal Hospital, Birmingham. A provisional scheme has been drawn up to rebuild the institution at a cost of £30,000. The latest annual report revealed a deficit of £811, and since the Insurance Act the number, both of in-patients and of operations, has increased. Moreover, the hospital does a considerable amount of work in treating surgical tuberculosis, and its present quarters are admittedly inadequate and badly equipped. How necessary proper equipment is, and how wide the range which modern science is opening to the orthopædist, the article alluded to makes sufficiently plain. We hope that it will be taken to heart by the Birmingham public, for whom we have pointed the moral in this Note.

### WOMEN DOCTORS AND MENTAL HOSPITALS.

In most branches of the hospital service, Poor Law and voluntary included, the difficulty of obtaining suitable resident medical officers has been the women doctors', or, more correctly, doctresses', opportunity. It is worth while noting, therefore, the tribute which Dr. Robert B. Campbell, medical superintendent of the Stirling District Mental Hospital, pays in his report to a recently left doctress on his assistant staff. "Dr. Isabella O. Emslie, who acted as third assistant medical officer and pathologist for over two years," he writes, "resigned on her appointment as one of the resident physicians at the Edinburgh Royal Hospital for Children." Then he adds this striking sentence: "Besides being an enthusiastic worker in our laboratory, where she did genuinely good work, Dr. Emslie was a first-rate medical officer and a great favourite with the patients." Women are not supposed, by Sir Almroth Wright and the rank and file of laymen who take refuge under his distinguished banner, to be endowed with much faculty for research. That is enough to make a medical man's tribute to a medical woman noteworthy. But when we remember that research work in mental hospitals is generally no more, and as little, as the enthusiasm of the A.M.O.s choose to make it, the case for women as mental residents becomes interesting as a possible improving step upon the road.

### THE ORDER OF MERIT.

The death of Dr. Alfred Russel Wallace reduces to fifteen (excluding three Japanese warriors) the membership of the Order of Merit, the highest distinction which the Sovereign can confer upon a subject. The late naturalist was not a member of the medical profession, but it is noteworthy that at the present time not a single medical man holds this decoration. Lord Lister had it, Sir Joseph Hooker had it (though not for eminence as a doctor), and the institutional world supplied its own special member in Miss Nightingale; but these three have all died within the last three years. We should refuse assent to a suggestion that medicine or any other science or profession is entitled to permanent representation. But we do say that at the present time the services of medical science to the community are such that they should be recognised in the membership of the Order of Merit. We have also a very definite idea as to the man we should select for this honour. To name him might be indiscreet; but we may hint that he is the *doyen* of a comparatively new branch of medicine, and that his services to humanity at large were especially and publicly acknowledged by our foreign guests at the time of the recent Congress of Medicine.

### CANCER RESEARCH AT MIDDLESEX HOSPITAL.

The twelfth Report of the Cancer Research Laboratories of the Middlesex Hospital (being Volume XXX. of the Archives of the Hospital) is remarkable for the fact that almost all the eighteen papers it contains deal with radium therapy for malignant disease, or with some of the properties of radium investigated to elucidate its therapy. Practically the whole energy of the staff of the Research Laboratories seems to be now directed upon radium—a concentration which at least shows organisation and discipline in the Laboratories even if it does not seem as yet to have produced much in the way of practical results. Indeed, to the casual clinician, a brief glance at this Report is calculated to give the impression that the Laboratories have got too much divorced from malignant disease clinically; for the protocols of experiments and researches are expressed in symbols so abstruse that they are incomprehensible to any but radium experts. We incline to the opinion that the entire detachment from practical radium therapy which the Research Laboratories seem to cultivate is injudicious.

### AN OUT-PATIENT CHAPLAIN AND HIS WORK.

The death of Mr. Arthur Hansell has deprived Norwich, and peculiarly the deaf and dumb patients in the diocese, of a man who made the visiting of this class of sufferers a fine art and a life's work. For fourteen years, since the late Bishop of Thetford founded the Diocesan Mission to the Deaf and Dumb, he was an indefatigable worker, a kind of out-patient chaplain, if we may use the word, who raised the routine of a chaplain's duties to its true high level of honourable and useful work. At the time of his death, to quote a tribute in a local paper, there were 400 deaf mutes with whom Mr. Hansell kept personal touch, and the centres which survive him at Ipswich, Yarmouth, Lowestoft, and Norwich (whence the movement began) are left intact now he is gone. Services, Bible-classes, and social entertainments all formed part of the work which he organised, and as "interpreter" at "a baptism, a confirmation, a wedding, or in the police court" he was always the invaluable friend and medium between those who live in sense-isolation and the world. Hospital chaplains in search of an ideal could do no better than study the aims and methods of Arthur Hansell.

### WHERE ARE YOUR BRAINS?

Nothing is more astonishing than the ignorance of people concerning the systems of which they form a unit, whether politically, economically, or institutionally. As no man can rise in his profession, however, who is not intellectually on the top of, instead of underneath, the system and professional details of his work, our Institutional Worker Supplement publishes each month questions, for the best answers to which money prizes are paid. The conditions attaching to the Question Column are described on page 2 of the Institutional Worker. A man who can answer the questions suggested knows his work, and, no less important, the work of his colleagues. A man who cannot answer them can learn enormously by attempting, and failing. Just "as activity is the only road to knowledge" so the prizewinner in these voluntarily offered contests of practical skill—all the questions deal with practical points—is the man who has attempted many things—the questions among them. The two asked for November concern the Number and Cost of surgical dressings used in the wards; and, especially for infirmary and mental hospital readers, the Method adopted in recording the receipt and issue of stores. The minimum payment for each published answer is half-a-guinea.

### LONDON CLUBS IN TIME OF WAR.

The much-paragraphed comprehensiveness of the Royal Automobile Club at the time of its opening will be turned to a new use in time of war, and include an unexpected form of accommodation, if Dr. H. E. B. Bruce-Porter's scheme is loyally acted upon. At a meeting of the club last week, he urged, on the outbreak of war, in which the Territorial Forces would be called out, hospital accommodation would be no easy task for them in London. Two resolutions were thereupon passed: That, in the above circumstances, the committee think it desirable that the Royal Automobile Club be handed over to the London County Territorial Association and other analogous bodies to be a general hospital, and this committee pledges itself to do everything in its power to secure the carrying into effect of this resolution. Secondly, that the R.A.C. be No. 3 General Hospital, as that hospital is commanded by Dr. H. E. B. Bruce-Porter, a member of the committee of the club, who would therefore be in touch with the R.A.C. staff. The importance of making arrangements beforehand is obvious. It would be a new, and, we hope, successful, undertaking for the Territorials in need of it. But can a club and a hospital be carried on in the same building at the same time? If the club has to be sacrificed temporarily, was such an emergency as is adumbrated prepared for in its rules? If not, how about the members, and the proverbial sticklers for the letter of their bond?

### THE PUBLIC AND POOR-LAW INFIRMARIES.

Once more the common case of "no beds" leading to the sending of a patient away from a voluntary hospital to a Poor-Law infirmary reveals the pressure from public opinion under which the existing Poor Law, commissions or no commissions, is forced to change. The present instance happens to be that of the North Staffordshire Infirmary, the crowded nature of which has led the Rev. E. H. Rogers, at a meeting of the Stoke-on-Trent Guardians, to ask if a woman in need of an operation, and the wife of "a respectable working man," under the regulations could be admitted to the Poor-Law infirmary. The Clerk replied that "destitution" was still technically and legally the only passport to admission, but he supposed that the present case was destitute in the sense of being unable to pay for suitable treatment in her own home. Mr. Rogers hinted that success in this case would lead to similar applications, and the Rev. H. S. Langley added the remark that the Poor-Law institution's theatre was a fine one, and might be used while accommodation was lacking at the voluntary hospital. It was a good, but inevitable suggestion, for, though the strictest letter of the law would not admit such cases, public opinion simply will not tolerate the turning away of cases from a publicly supported institution. Thus the law is altered, happily, without Act of Parliament, and the germ of a future hospital system continues to grow.

### DIFFERENCE OF OPINION ON THE MENTAL DEFICIENCY ACT.

On the London County Council a difference of opinion has arisen with reference to the administration of the Mental Deficiency Act. In appointing a statutory committee for the purposes of the Act there are three courses open to the Council: To constitute a new committee consisting partly of members of the Council, and partly of persons (including women) appointed from outside the Council, the members of the Council to form a majority of the committee; to appoint the existing Mental Hospitals Committee with the addition of at least two women to act as the committee for the care of the mentally defective; and, thirdly, to constitute a new committee consisting of all the members of the Mental Hospitals Committee, with addition of persons (including women) appointed from outside the Council as in the case of the first alternative. The General Purposes Committee, the Education Committee, and a Special Committee suggest that the first of the three courses should be followed, and all the mental hospital work should be transferred to the new committee, it being urged that by this means persons appointed from outside the Council, having special knowledge and experience, would be able to deal with work arising under the Lunacy Acts as well as that arising under the Mental Deficiency Act. The Mental Hospitals Committee, however, do not agree with this view, and urge that the existing committee should be employed on the work. They point out that the Act deals only with limited classes of the mentally defective, that they are already dealing with other classes, and that they already have in their service a staff of officers with special knowledge and administrative experience. They are also strongly of opinion that the introduction of co-opted members would not be to the advantage of the administration of the mental hospitals.

### LONDON AND ITS AMBULANCE SERVICE.

A few weeks ago, as stated in The Hospital of October 25, Major Gray, chairman of the General Purposes Committee of the London County Council, said that he had no doubt that under the powers recently granted them by Parliament the Council would be able to provide an effective and efficient ambulance service for the whole of London, and that, he trusted, before the close of the current year. We are afraid, however, from later developments, that this optimistic forecast has little chance of being realised. As a first step towards obtaining an ambulance service the General Purposes Committee informally approached the Metropolitan Asylums Board, which, having in its possession a large number of ambulances, appeared to be the first authority which ought to be consulted in the matter. As a result of the conference the committee now propose to invite the Asylums Board to prepare and submit to the Council at the earliest possible date a detailed scheme, with estimates of cost, of co-operation with the Council and other authorities in the establishment in London of an efficient ambulance service for street accidents. The committee say that they have reason to believe that this could be achieved in time for its consideration by the Council "before the end of the current year," but the actual ambulance service seems to be still a remote prospect. This is evidently the view of the Progressive members of the Council, who are urging that a special committee should be appointed to consider and report forthwith as to the best methods by which the Council itself can organise an ambulance system on the lines of that worked by the City Corporation.

### THE PAY OF INFIRMARY SCRUBBERS.

There are few employees in hospitals and infirmaries who have less consideration shown to them than the scrubbers. Their hours are long, and the pay low. As a rule in infirmaries they are women in adverse circumstances, whom Guardians keep off the rates by finding them this work. The Wandsworth Board of Guardians, who have been the pioneers in many reforms in infirmaries, have adopted a scheme reducing the hours of their labour to 48 a week, whilst the wages have been increased from 2*s.* 6*d.*, the usual sum paid in nearly all hospitals and infirmaries, to 2*s.* 10*d.* a day, whilst they receive 2*s.* for half a day's work on Sundays. The concessions are slight, but may mean much to the women, many of whom have children to support, and the extra liberty at night will permit them to give more time and attention to their families. The reduction in hours has led to extra women being engaged, as it was found that the whole of the work could not be completed in the limited time. Whether the Local Government Board will agree to the alterations remains to be seen, but as a rule that body is none too generous when dealing with minor officials.

### MEDICAL POLICY AND SANATORIUM PATIENTS.

At a meeting of the Staffordshire Insurance Committee last week grave charges, including one of drunkenness, were preferred against the patients at Moxley Sanatorium. Dr. Shufflebotham stated that drinking by patients had occurred at a neighbouring public-house; that rare cases of actual drunkenness at the sanatorium itself had been remarked. Nothing, he added, is worse than alcohol for consumptives. Mr. Webb, chairman of the sanatorium committee, in reply, said that the committee knew that there had been drinking, and, far from winking at it, had discharged one patient for the offence on its discovery. He added that there was no cause for alarm, and undertook that a stringent rule should be made against patients entering the public-house. So far, so good; but this is only the latest proof of the need for expert training in the administration of sanatoria and in the management and control of adult semi-convalescents like consumptives. In private conversation the other day, a very highly placed official with the bestowal or selection of applicants for these posts within his immediate purview, if not actual control, told us that men with experience of sanatorium management in this country were practically unobtainable, hence the unsatisfactory appointments. We invite our readers to challenge this statement by sending us their views upon this question.

### GUY FAWKES DAY AND A GRATEFUL PATIENT.

Twelve months ago a patient was admitted to the Walthamstow Hospital suffering from severe burns occasioned by making fireworks in preparation for November 5 for his son, in which process the ingredients exploded, injuring the boy as well and necessitating also his admission as an in-patient. Both patients, however, we are informed, made good recovery, and it appears that the father is the secretary of a local choral society, with the result that we now learn that a series of concerts is being organised by him in aid of the hospital out of gratitude for the kindness shown him. Two such concerts, we understand, have already been held.

### A PROPOSED MEDICO-PSYCHOLOGICAL CLINIC.

A meeting was held in London last week to inaugurate a practical alliance between general psychology and psychotherapy, so that both can be carried on in one institution. The committee in charge of the scheme, of which Mrs. Hall, 41 Adelaide Road, Surbiton, is the acting secretary, desire a quiet site for the building close to University College, since Professor Spearman, of that institution, is their consulting psychologist. The committee, in appealing for funds, point out that only at Liverpool is there in this country a clinic for psychotherapeutic treatment, and believe that, while retaining control of the organic side of their patients' diseases, they will welcome an opportunity for investigating the psychic side—presumably in the psychological laboratory of the proposed clinic. If and when the appeal for money is successful, it will be time enough to criticise the institution. At present it labours under the disadvantage of dealing with a still so largely inexact branch of science—a disadvantage that will make its successful inauguration even a matter of difficulty.

### THE RADIUM INSTITUTE.

Radium seems determined to keep in the public eye just at present. In the latest instance it has got into the courts as well, or rather brought the British Oxypathor Company there. Sir Frederick Treves and Mr. A. E. Hayward Pinch, F.R.C.S., the medical superintendent of the Radium Institute, and others connected with it, sued the above Company, trading as the Radium Oxygen Institute, and obtained an injunction restraining the defendants from employing the words "Radium Institute," or advertising in such a way as likely to mislead the public into thinking that the defendants' business was the same or connected with the well-known institution founded by Sir Ernest Cassel and others. The defendants also undertook not to infringe the Radium Institute's copyright in a publication entitled "A Report of the Work carried out at the Radium Institute from August 4, 1911, to December 31, 1912." Once the public imagination has been excited concerning the "curative" properties of any new substance or drug, indiscreet commercial undertakings always have a happy hunting-ground for a time by such trespass as the above action was taken to stop. The protection of the public, in these matters, is also the protection of science.

### THE REBUILDING OF MELBOURNE HOSPITAL.

At the Melbourne Hospital, writes our Australian correspondent, the new buildings are progressing satisfactorily. New quarters for 170 nurses have been occupied for some months; the out-patients' department is finished, but not in use until the casualty block is ready; the medical block and surgical block—six wards in each—are finished and now occupied.

### THIS WEEK'S DRUG MARKET.

No improvement is as yet noticeable in the market for drugs, and price changes of importance have been few during the week. A fair business is being done in opium at practically unchanged prices, but in the primary market there is a tendency towards higher prices. Quinine is quiet, and there is no change in makers' prices. Morphine and codeine are unchanged in price. Eucalyptus oil is unaltered in value, but it is probable that any alteration would be in an upward direction. The demand for cod-liver oil is still very dull, and prices have a lower tendency. There have been reductions in the prices of aloin, apomorphine, and emetine.

